# Author Correction: Loss of MTX2 causes mandibuloacral dysplasia and links mitochondrial dysfunction to altered nuclear morphology

**DOI:** 10.1038/s41467-020-19290-y

**Published:** 2020-10-19

**Authors:** Sahar Elouej, Karim Harhouri, Morgane Le Mao, Genevieve Baujat, Sheela Nampoothiri, Hϋlya Kayserili, Nihal Al Menabawy, Laila Selim, Arianne Llamos Paneque, Christian Kubisch, Davor Lessel, Robert Rubinsztajn, Chayki Charar, Catherine Bartoli, Coraline Airault, Jean-François Deleuze, Agnes Rötig, Peter Bauer, Catarina Pereira, Abigail Loh, Nathalie Escande-Beillard, Antoine Muchir, Lisa Martino, Yosef Gruenbaum, Song-Hua Lee, Philippe Manivet, Guy Lenaers, Bruno Reversade, Nicolas Lévy, Annachiara De Sandre-Giovannoli

**Affiliations:** 1 Aix Marseille Univ, INSERM, MMG, U1251 Marseille, France; 2 MitoLab, Mitochondrial Medicine Research Centre, Institut MITOVASC, CNRS UMR 6015, INSERM U1083, Université d’Angers, CHU d’Angers, Angers, France; 3 Department of Medical Genetics, INSERM UMR 1163, Paris Descartes-Sorbonne Paris Cité University, IMAGINE Institute, Necker Enfants Malades Hospital, Paris, France; 4 Department of Pediatric Genetics, Amrita Institute of Medical Sciences & Research Centre, AIMS Ponekkara PO Cochin, Kerala, India; 5 Medical Genetics Department, Koç University, School of Medicine (KUSoM), Istanbul, Turkey; 6 Neurology and Metabolic Division, Cairo University Children Hospital, Cairo, Egypt; 7 Medical Genetics Service Specialties Hospital FF AA No.1, Quito, Ecuador; 8 Institute of Human Genetics, University Medical Center Hamburg-Eppendorf, Hamburg, Germany; 9 Pole of Anesthesiology and Reanimation, , Necker Enfants Malades Hospital, Paris, France; 10 Department of Genetics, Institute of Life Sciences, Hebrew University of Jerusalem, Jerusalem, Israel; 11 Centre National de Recherche en Génomique Humaine (CNRGH) and Centre d’Etude du Polymorphisme Humain (CEPH), Institut de Biologie François Jacob, CEA, Université Paris-Saclay and Fondation Jean Dausset, Paris, France; 12 INSERM UMR1163, Institut Imagine, Paris, France; 13 CENTOGENE AG, Rostock, Germany; 14 Institute of Medical Biology, A*STAR, Singapore, Singapore; 15 Sorbonne Université, INSERM, Institute of Myology, Center of Research in Myology, Paris, France; 16 CeleScreen SAS, Paris, France; 17 APHP, Biobank Lariboisière BB-0033-00064, Platform of BioPathology and Innovative Technologies in Health, Hôpital Lariboisière, Paris, France; 18 INSERM UMR1141 « NeuroDiderot », Université de Paris, Paris, France; 19 Department of Medical Genetics, La Timone Children’s Hospital, Marseille, France; 20 Biological Resource Center (CRB-TAC), Assistance Publique Hôpitaux de Marseille, La Timone Children’s Hospital, Marseille, France

**Keywords:** Apoptosis, Energy metabolism, Next-generation sequencing, Nuclear envelope, Genetics research

Correction to: *Nature Communications* 10.1038/s41467-020-18146-9, published online 11 September 2020

The original version of this Article contained errors in the author affiliations.

The affiliations of Nathalie Escande-Beillard and Bruno Reversade with ‘Medical Genetics Department, Koç University, School of Medicine (KUSoM), Istanbul, Turkey’ were inadvertently omitted.

This has now been corrected in both the PDF and HTML versions of the Article.

