# Supplementary material for: Loss of MTX2 causes mandibuloacral dysplasia and links mitochondrial dysfunction to altered nuclear morphology
Source: Nat Commun. 2020 Sep 11;11:4589. doi: 10.1038/s41467-020-18146-9 (PMC7486921; doi:10.1038/s41467-020-18146-9)
Supplement: Supplementary file 1 — Supplementary Information [file 41467_2020_18146_MOESM1_ESM.pdf]

SUPPLEMENTARY INFORMATION

Loss of MTX2 causes Mandibuloacral Dysplasia and links mitochondrial dysfunction to altered nuclear morphology

Elouej et al.

SUPPLEMENTARY NOTE 1: Patients' clinical features

We evaluated 7 patients from different geographical origins of whom 3 were related, all from ascertained or suspected consanguineous unions, showing hallmarks of severe progeroid mandibuloacral dysplasia. Four of them (MADM1, 2, 3, 5) were sporadic cases without family history, while MADM4-1 to 3 were related. The clinical features and some radiological features of the patients are presented in Figure 1, Supplementary Figure 1, Supplementary Figure 2, Supplementary Figure 3 and Supplementary Data File. All of them showed severe mandibular hypoplasia, a long nose with small viscerocranium and preservation of fat in the cheeks, prominent eyes, small mouth, generalized lipodystrophy, weight and head circumferences <P1, severe postnatal growth retardation (starting by 24 mo), atrophic skin with hyper/hypopigmentation, distal acro-osteolysis of phalanges of fingers and toes and hypoplastic clavicles. Three patients presented with focal segmental glomerulosclerosis, all patients excepted MADM5 presented with extremely severe hypertension. Of note, none presented with hearing loss and psychomotor development was normal for all.

The Indian girl patient (**MADM1**) was first seen at the age of 4 years and had been followed up for progressive glomerular disease since her 2nd year of age, complicated with hypertension and severe proteinuria. Her blood pressure was controlled with antihypertensive medications. The renal biopsy features were suggestive of focal glomerulosclerosis with crescents (4%). She presented with classical features of acroosteolysis of hands and feet with nail dystrophy, severe mandibular recession and distal clavicular hypoplasia. She presented with poikiloderma, sparse body hair and hyperkeratosis of palms and soles. At 11y she had a height of 99 cm (<P1), weight of 13.5 Kg (<P1), head circumference of 45.5 cm (<P1).

The Turkish boy (**MADM2**) was first seen at the age of 14 years. He was reported as having normal appearance in the first 2 years of life upon the photos that were presented (Figure 1). The family stated that he began to show stunted growth thereafter leading to severe growth retardation, atrophic and translucent skin with mottled pigmentation and generalized loss of subcutaneous fat. At the age of 14 years his height was 112 cm (<P1), weight 16.5 kg (<P1) and head circumference 48,2 cm (<P1). Radiographs showed acroosteolysis with brachydactyly more pronounced distally and clavicular hypoplasia, soft tissue calcification and patent anterior fontanel. The patient had malignant hypertension and mitral valve prolapsus, leading to insufficiency and stenosis. Renal biopsy was performed due to severe proteinuria (2gr/day). Major morphologic changes were observed mainly in the arterial vascular compartment, showing significant fibrointimal thickening with multilamellation and fragmentation of the internal elastic lamina. Arterioles revealed hyaline changes and mild myointimal hypoplasia. Glomeruli had nonspecific ischemic alterations, focally in the form of glomerulo-basement membrane wrinkling, thickening and splitting. Immunofluorescence microscopy was negative for immune complex deposition. Thorax CT showed enlarged left ventricle of the heart and thickened interventricular septum (13 mm), enlarged truncus pulmonalis (28 mm) and focal calcifications of coronary arteries. Intravascular structures were prominent in the lungs. Mosaic perfusion pattern at basal regions of the lungs and fibroatelectasic regions reaching the pleura were noted. All biochemical parameters were in normal range, except ALP (alkaline phosphatase), AST and vitamin D. No results were suggestive for diabetes and insulin resistance. There was no hyperlipidemia and hormone levels were in normal ranges. The patient deceased due to heart failure at the age of 17.5 years.

The third patient (**MADM3**) is an Algerian young boy who was referred for molecular investigations at the age of two and a half years. His developmental milestones were normal until one year of age, when he began to show stunted growth, progressive loss of scalp hair and eyelashes, lipodystrophy and ectodermal abnormalities with dystrophic nails, amelogenesis imperfecta and diffuse cutaneous hypopigmented papules (Figure 1, Supplementary Figure 1 and Supplementary Data File). At 7 months, after an episode of severe viral bronchitis, he presented with marked hypotonia which impaired his nutritional state and required gastrostomy. He developed recurrent pulmonary infections leading to chronic respiratory insufficiency with the need of continuous non-invasive ventilation, as well as recurrent episodes of acute renal insufficiency upon viral infections. The first episode was though due to a severe hypertension peak at 1 year of age, which allowed to diagnose his hypertensive condition; the last, at age 6 years, featured acute tubular necrosis. The child developed hepatosplenomegaly since the first months of life and had a very high-pitched voice due to vocal cords hypoplasia and dysfunction. Further investigations showed cardiac anomalies (mitral insufficiency and left ventricular hypertrophy) associated with severe hypertension needing multi-drug regimen. He had a good cognitive development. At the age of 7 he was hospitalized for seizures related to a major hypertensive encephalopathy (Pas = 190 mmHg) associated with PRESS syndrome. Cerebral MRI showed stroke-like features. CT angiography showed a mid-aortic syndrome with an abdominal aorta of 2 mm diameter, very small renal and mesenteric arteries and multiple vascular arteriosclerotic calcifications. Patient MADM3 also presented severe osteoporosis leading to recurrent fractures, coxa valga with dislocation of the femoral heads and distal acroosteolyses. Routine biochemical test revealed dyslipidemia.

MADM4-3, the proband, the second child of first-degree cousins of Egyptian origin, was seen due to delayed achievement of developmental milestones. He was born by cesarean section, had cyanosis at birth and was hospitalized at intensive care unit for 5 days, receiving surfactant therapy. Gross motor developmental delay was noticed since he acquired head control at the age of 5 months and walked at 18 months. At the age of 9 months, he had an episode of tonic seizures for which he received antiepileptic treatment. Patient has also delayed language development. He experienced recurrent pulmonary infections. Echocardiography revealed mitral valve prolapsus, moderate mitral regurgitation and mild left ventricular hypertrophy. The patient received diuretics and ACE inhibitors for hypertension. Clinical examination revealed facial dysmorphism (Figure 1, Supplementary Figure 1 and Supplementary Data File), brachycephaly, progressive loss of hair which was brittle and hypopigmented, sparse eye-brows, dystrophic nails, generalized hypotonia with brisk reflexes, cardiomegaly, hepatomegaly, exceeding 5 cm the costal margin. Laboratory investigations revealed increased serum transaminases, abdominal ultrasonography revealed hepatomegaly with coarse texture and macrovesicular steatosis. Brain MRI showed bilateral periventricular hyperintense T2 changes with posterior predominant distribution and mild ectasia of the posterior horn of the lateral ventricles.

MADM4-1 and 2 were two brothers born to consanguineous Egyptian parents and were first degree cousins of MADM4-3 (Figure 1, Supplementary Figure 1 and Supplementary Data File). **MADM4-1** suffered from hyperthermia, severe intractable diarrhea with progressive progeroid appearance, loss of hair, nail dystrophy and hypodontia. He died at the age of 2 years and 2 months.

MADM4-2 presented at the age of 7 months with a similar clinical picture as MADM4-3, with global developmental delay, generalized hypotonia, gradual progressive loss of hair, skin and hair depigmentation, progeroid facies, dystrophic nails, hepatosplenomegaly with vesicular hepatic steatosis, abnormality of the femoral epiphysis, and white matter hyperintensities on brain MRI.

Patient MADM5 was the first child of healthy unrelated parents from Ecuador (Supplementary Figure 3). Pregnancy was complicated by oligohydramnios at the end of pregnancy leading to birth by cesarean section at 39 weeks of gestation with APGAR scores of 8 and 9 at one and five minutes, respectively. His birth weight was 3500g (51P), length 46 cm (<P1) and he had a normal occipitofrontal head circumference (OFC) (35 cm, 41P). A patent foramen ovale was observed at two years without clinical repercussion. At the clinical evaluation at the age of 1 year and 5 months he had a triangular face, sparse scalp hair, thin and translucent skin with prominent veins, frontal bossing, large eyes, low-set ears and mandibular underdevelopment. Moreover, he had bilateral cryptorchidism, global lipodystrophy, joint hypermobility, nail hypoplasia, and had developed an inguinal hernia. All milestones of motor development were normal: rolling over at 6 months, sitting at 8 months, and walking at fourteen months. Language development wasn't delayed. Clinical evaluation at the age of 3 years confirmed previous facial dysmorphic findings and revealed a short philtrum. He had brittle teeth, requiring multiple dental restorations at age 4 years, and some secondary teeth were absent. A brain CT, performed at the age of 2 years, was normal. At the age of 5 years, he had two bone fractures, one in left femur and another one at rib. In addition, the thin-appearing, transparent and wrinkled skin with prominent venous network persisted. He developed bilateral corneal opacities. At the age of 6 years muscular hypotonia with decreased muscle mass became prominent, together with hypoplasia of the nasal

wings, and very sharp voice. His weight has constantly been 10 kg from 3 years to 6 years of age, where height was 85 cm (<P1). Echocardiogram was normal, as well as hormonal, lipid and renal profiles. There was no hypertension nor glomerulosclerosis at 6 years of age. The skeletal abnormalities are genu valgum (Supplementary Figure 3). Radiological studies showed delayed appearance of the growth nucleus of the wrist and reduced bone densities. Hips partially failed to cover femoral heads, however acetabula angles were adequate. Auditive responses and neurodevelopment were normal.

SUPPLEMENTARY FIGURES AND LEGENDS

Supplementary Figure 1

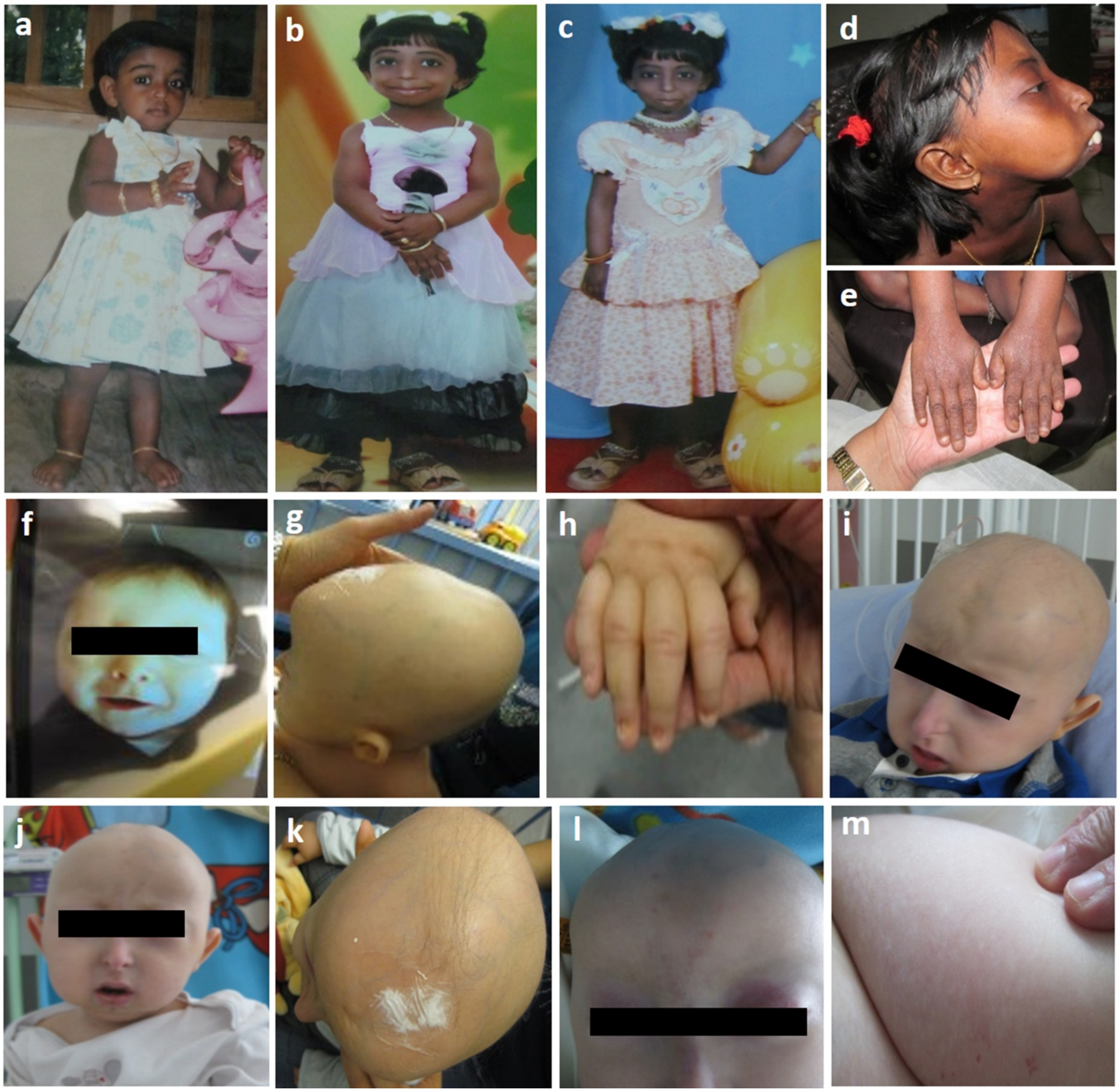

Supplementary Figure 1: further clinical characteristics of patients affected with MADaM Syndrome. (a-e) Pictures of patient MADM1 at different ages from 2 years with normal appearance **(a)**, 7 years **(b)**, 8 years **(c)** and 9 years **(d, e)** showing the disease progression

especially severe mandibular recession, lipodystrophy, pinched nose, long ears, a small jaw and nail dystrophy. **(f-m)** Pictures of MADM3 patient at different ages from 18 months with normal appearance **(f)**, 2 years **(g, h)** and 3 years **(i-m)** showing alopecia, frontal bossing, apparent veins, pinched nose, a small mouth, a small jaw, poikiloderma and nail dystrophy. Consent was obtained to publish patients' images.

Supplementary Figure 2

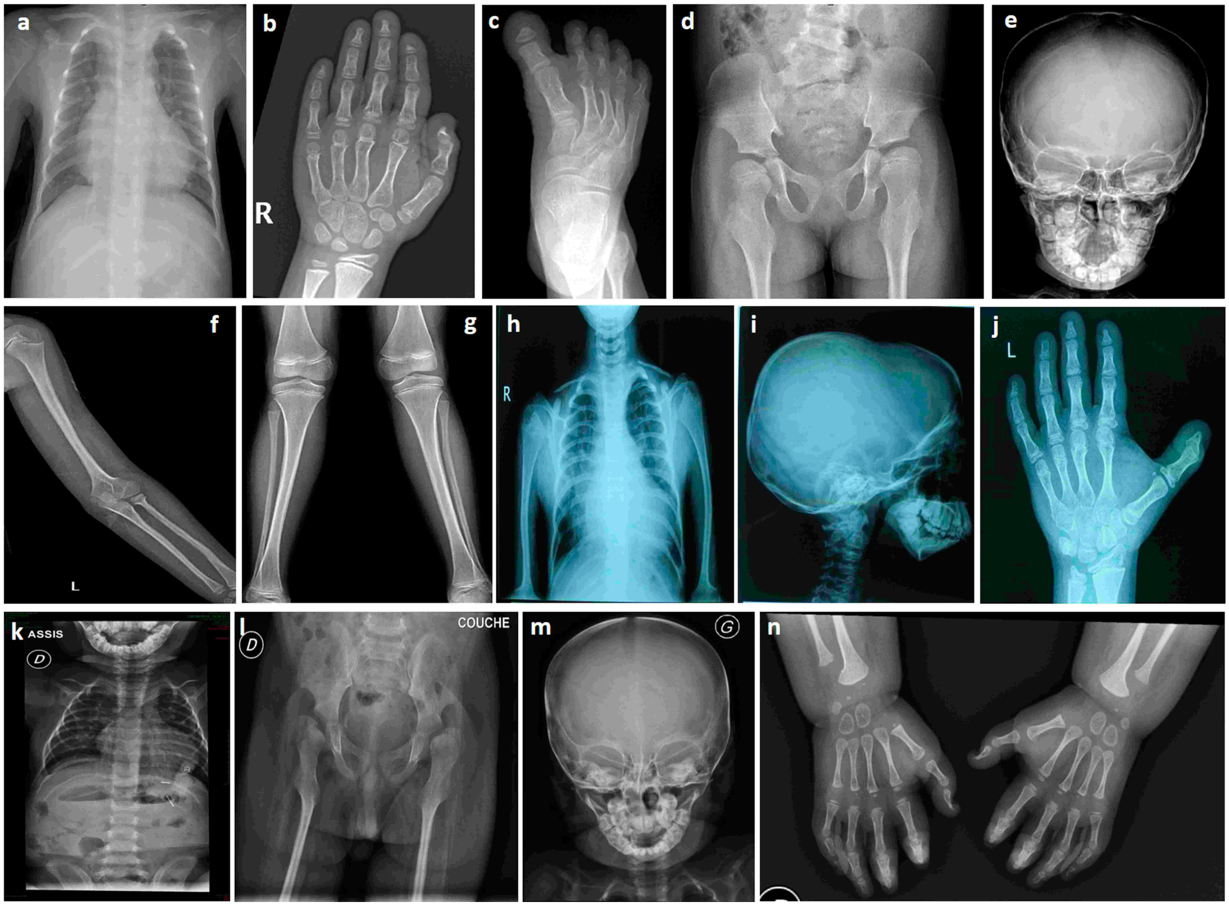

Supplementary Figure 2: radiographical features of patients MADM1 (a to g), MADM2 (h to j) and MADM3 (k to n). Chest, hand, foot, pelvis, cranium, upper and lower extremity radiographs of patient MADM1 showing **(a)** 13 ribs on the left, narrow thorax, thin clavicles with lateral one third deficiency, **(b)** carpal bone age corresponding to 7 years at chronological age of 9, short metacarpals and phalanges, acroosteolysis with prominent involvement of index finger and thumb, **(c)** brachydactyly of toes with short metatarsals and acro-osteolysis of left foot at 9 years of age, **(d)** tapering of ileum, bilateral flat femoral heads and short femoral neck, hypoplastic pedicles of S1, S2 with fusion on the left, **(e)** open anterior fontanel at 11 years, **(f)** thin humerus,

radius and ulna with narrow medullary cavity, **(g)** genu valgum, thin fibula and large lower femoral epiphysis. **(h-j)** Chest, lateral cranium and hand radiographs of patient MADM2, showing **(h)** bilateral distal clavicle hypoplasia as well as thin and curved humeral bones, **(i)** frontal, parietal and occipital bossing, patency of the anterior and posterior fontanelles, **(j)** lysis of the distal phalanges. **(k-n)** Radiographs of patient MADM3, showing **(k)** gracility of clavicles and thin ribs **(l)** hip dislocation with major coxa valga, broad and long femoral necks, enlarged metaphyses, and gracile femora, **(m)** open cranial sutures at 4 years, **(n)** acroosteolysis with hypoplasia of distal phalanges. Informed consent was obtained to publish patients' radiographs.

Supplementary Figure 3

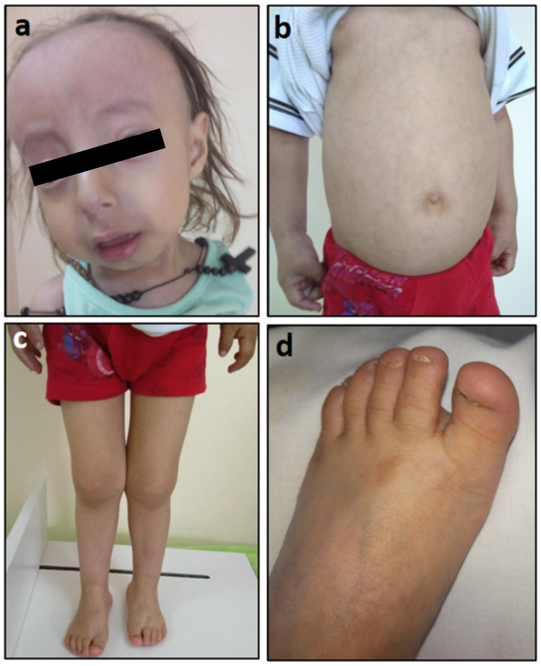

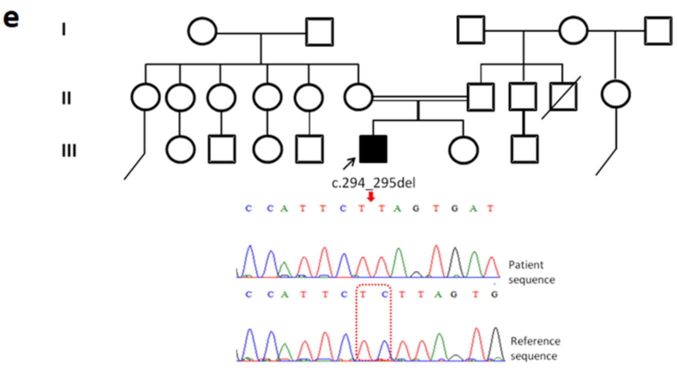

f

Chr	Start	End	Size (bp)	Type
2	173366456	178432094	5065639	ROH ⬅ Includes MTX2
6	107212061	110103315	2891255	ROH
12	40570404	43383532	2813129	ROH
11	72584486	75334940	2750455	ROH
5	40681401	43123153	2441753	ROH
20	47757443	49812971	2055529	ROH
11	85111764	86952254	1840491	ROH
2	110995872	112722037	1726166	ROH
9	128128781	129722342	1593562	ROH
14	101026211	102326049	1299839	ROH
1	1755523	3021701	1266179	ROH
19	42583332	43776828	1193497	ROH
12	108827237	109783826	956590	ROH
11	64990024	65833995	843972	ROH
4	38137235	38828194	690960	ROH
2	240967659	241572132	604474	ROH
9	37126704	37692529	565826	ROH
17	16948713	17491297	542585	ROH
19	49010092	49433528	423437	ROH
6	30186422	30583509	397088	ROH
12	57064274	57454785	390512	ROH
5	840813	1219744	378932	ROH
17	76019077	76385474	366398	ROH
22	22880890	23242945	362056	ROH
8	143379243	143730195	350953	ROH
12	132717645	132856654	139010	ROH
Total autosomal ROH			33 950 287,00	

Supplementary Figure 3: clinical features and genomic findings in patient MADM-5. (a-d) clinical features of patient MADM-5 at age 6, including **(a)** frontal bossing, retromicrognathism, short philtrum, sparse hair, thin skin with prominent superficial vessels; **(b)** thin skin with prominent venous network on the abdomen; **(c)** genu valgum; **(d)** nail hypoplasia; **(e)** pedigree of the patient's family, showing he's a sporadic case. **(f)** Regions of homozygosity (ROH) from the WES data of the patient are shown. Altogether ROH span 82 Mb (Megabases) of the patient´s exome. The largest autosomal ROH in chromosome 2, spanning 5 Mb (in red), contains the *MTX2* locus.

Supplementary Figure 4

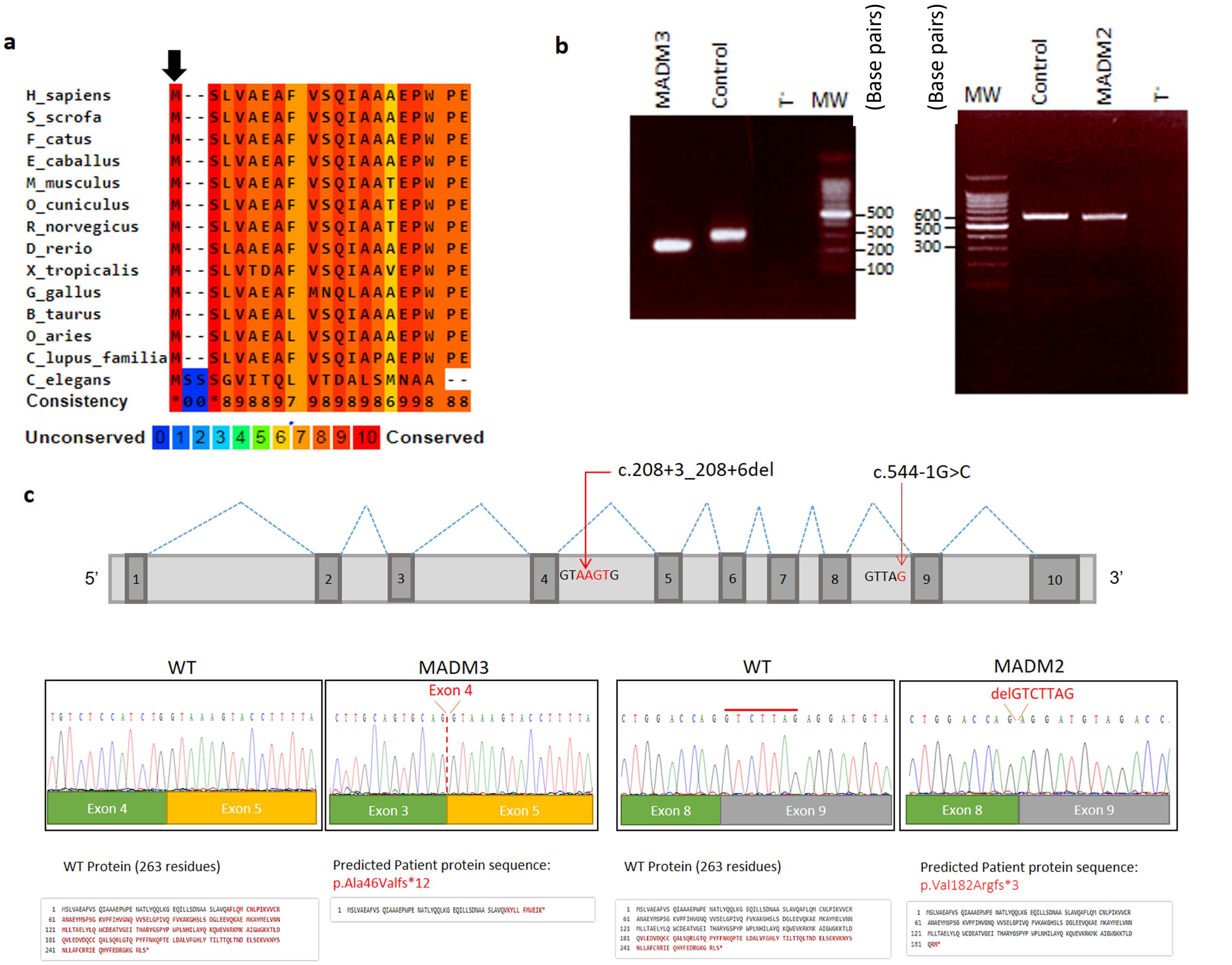

Supplementary Figure 4: characterization of MTX2 mutations. (a) Aminoacid sequence alignment of the first 20 residues of human MTX2 and its orthologs from the indicated species, showing the evolutionary conservation of the Met1 residue (indicated with an arrow). **(b)** Ethidium bromide stained agarose gel of RT-PCR amplified *MTX2* cDNA products from controls and patients MADM3 and MADM2 carrying splicing mutations. "T-" : PCR negative control (no cDNA); MW: molecular weight in base pairs. **(c)** Sanger sequence analysis of control and patients *MTX2* cDNAs and the predicted consequences on protein translation (lower panels).

Supplementary Figure 5

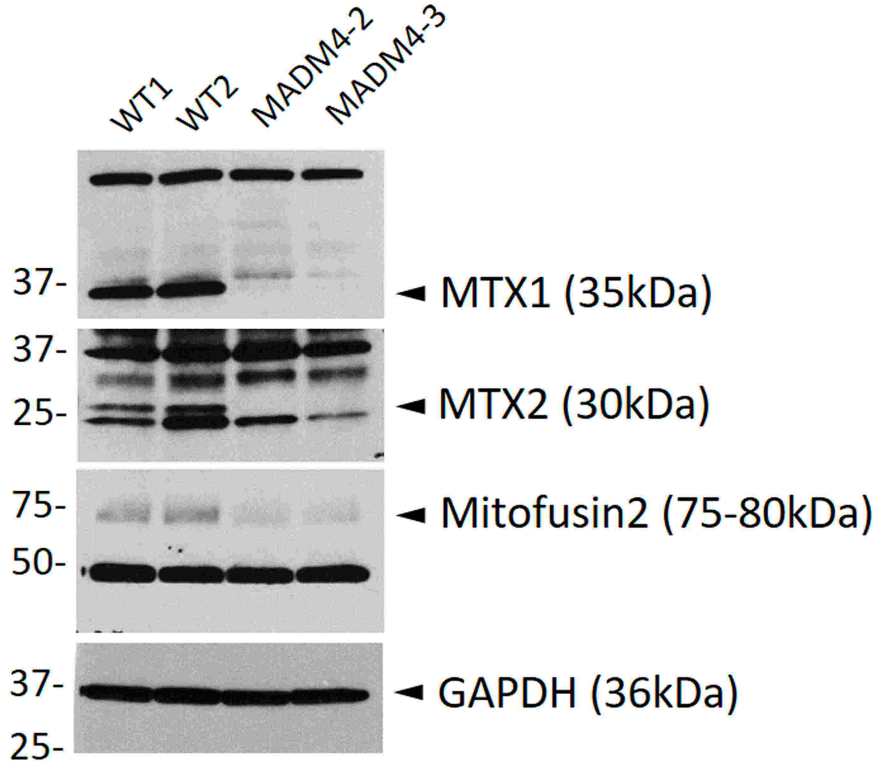

Supplementary Figure 5: Immunoblots from primary fibroblasts of patients MADM4-2 and MADM4-3. MTX2 and MTX1 are not detected in patients' protein lysates and Mitofusin 2 expression levels are reduced compared to control cells (WT1, WT2). The data shown is issued from one experiment. Source data are provided as a Source Data file.

Supplementary Figure 6

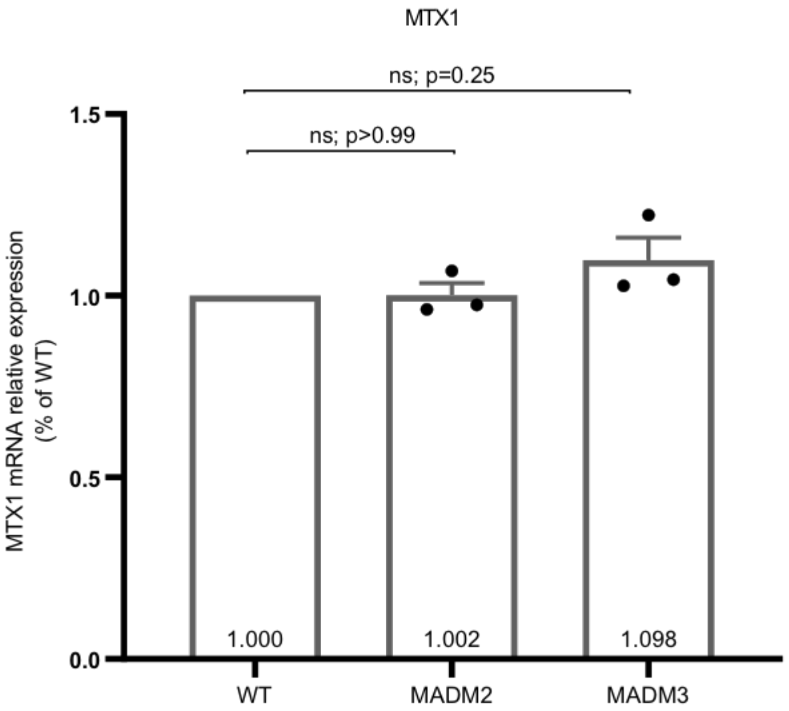

Supplementary Figure 6: Evaluation of *MTX1* transcripts' levels. Quantitative analyses of *MTX1* cDNA levels by qPCR in WT, MADM2 and MADM3 fibroblasts. Transcripts' levels were normalized to *RPS13* values and expressed as percent of WT, whose values were set to 1 for each experiment; n=3 independent experiments. Wilcoxon matched-pairs signed rank test, mean ± SEM are shown, with mean values indicated within bars; Lower 95% CI (WT: 1; MADM2: 0,8581; MADM3: 0,8294), Upper 95% CI (WT: 1; MADM2: 1,145; MADM3: 1,366); the exact *p*-values are indicated, ns=not significant. Source data are provided as a Source Data file.

Supplementary Figure 7

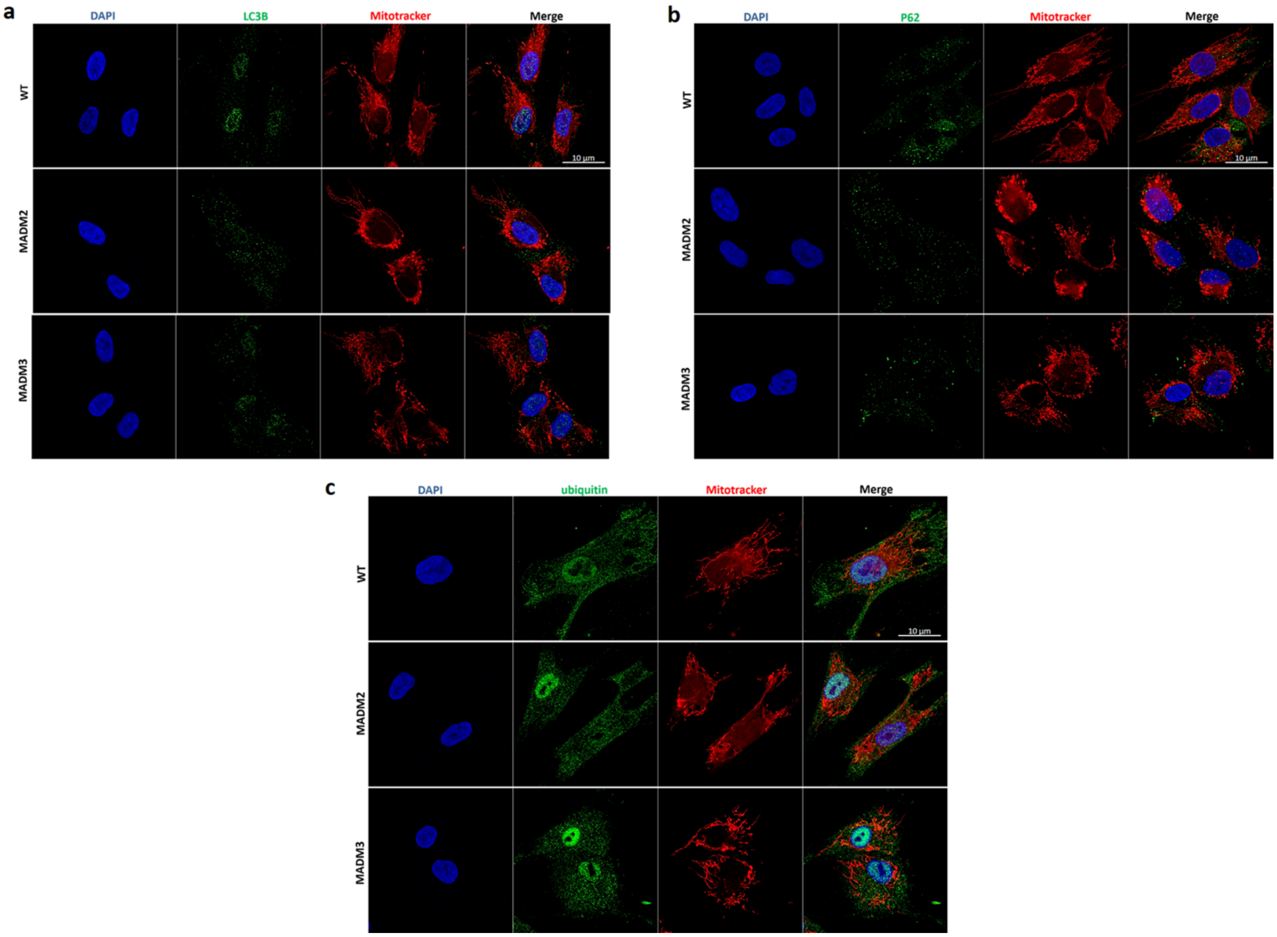

Supplementary Figure 7: the classical parkin/ubiquitin autophagic pathway involving ubiquitination, LC3B and P62 is not activated in patients' fibroblasts. Immunofluorescence staining of LC3B **(a)** P62 **(b)** and ubiquitin **(c)** (green) with Mitotracker (red) together with DAPI (4′,6-diamidino-2-phenylindole, blue), in patients' and control's fibroblasts. Neither ubiquitin nor LC3B nor P62 colocalized with the mitochondrial network in patients' fibroblasts. The data shown is representative of n=2 independent experiments for each condition. Scale bar, 10 µm.

Supplementary Figure 8

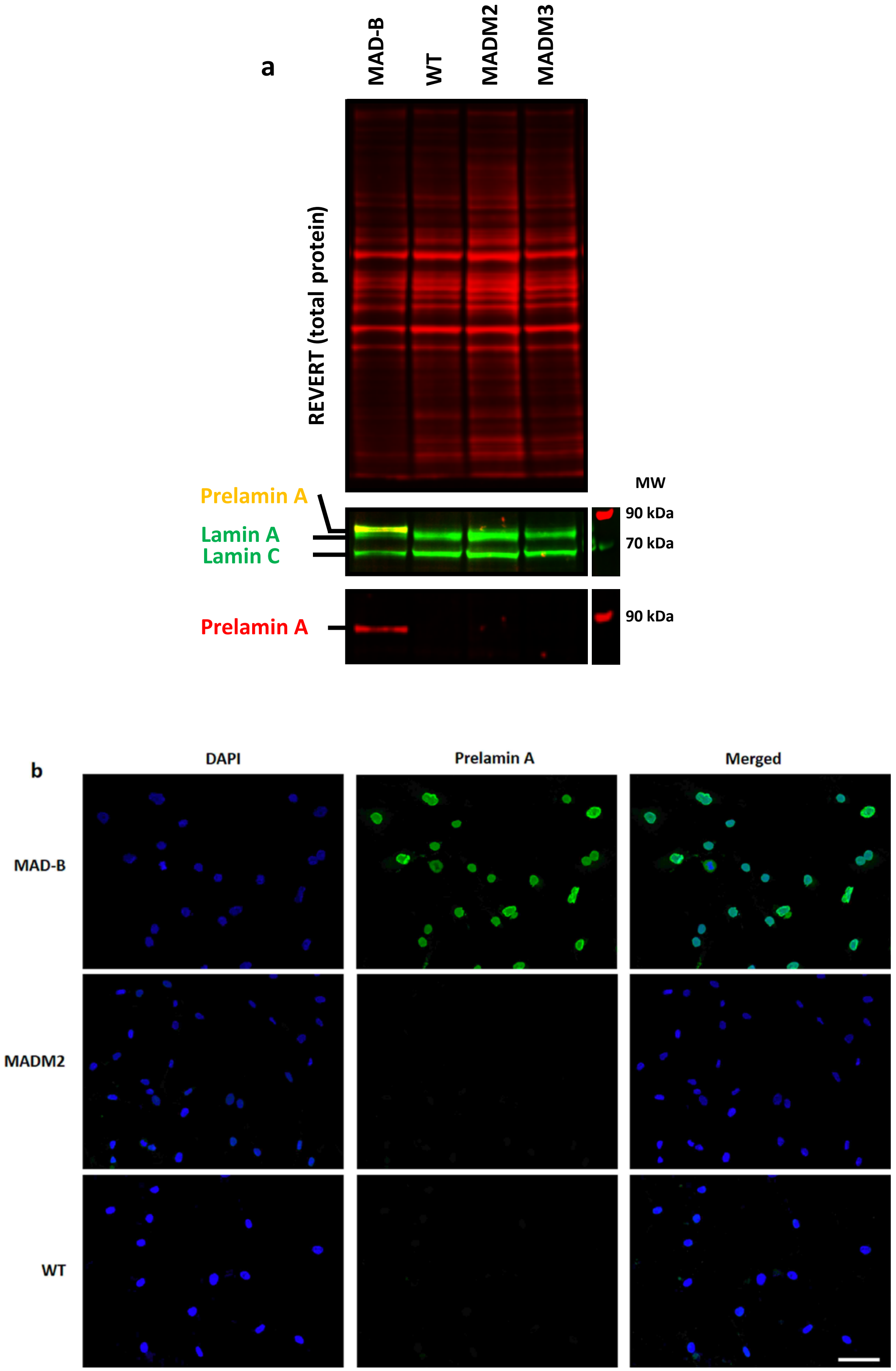

Supplementary Figure 8: evaluation of prelamin A expression. **(a)** A representative Western blot experiment in fibroblast whole cell lysates from healthy control (WT), MADM2, MADM3 and MAD-B patients, showing Prelamin A, Lamin A and Lamin C expression. Results are representative of n=3 independent experiments. Source data are provided as a Source Data file. **(b)** Prelamin A indirect immunofluorescence staining, together with DAPI, in MAD-B, MADM2 and a healthy control (WT) fibroblast cell lines at equivalent culture passages. Results are representative of one experiment. Scale bar: 40 µm.

Supplementary Figure 9

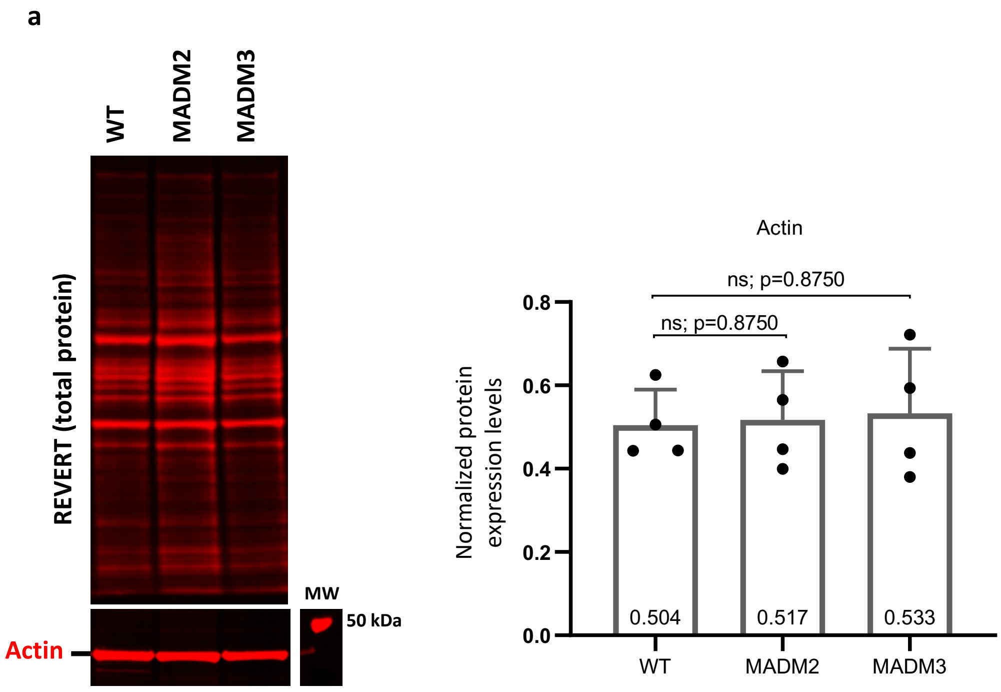

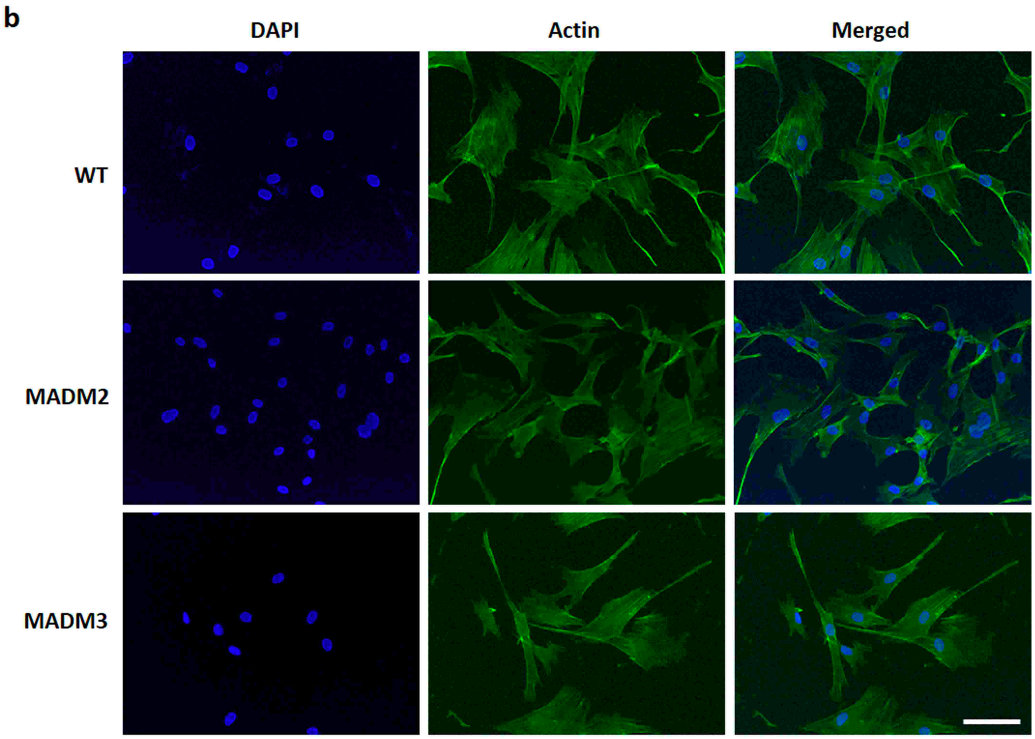

Supplementary Figure 9: evaluation of actin expression. (a) A representative Western blot experiment in fibroblast whole cell lysates from healthy control (WT), MADM2 and MADM3 patients, showing actin expression. Protein levels were quantified by ImageJ software and their expression levels were normalized to total protein (Revert staining). Results are expressed as mean ± SEM, n=4 independent experiments. Wilcoxon matched-pairs signed rank test, Lower 95% CI (WT: 1; MADM2: 0,5772; MADM3: 0,4869), Upper 95% CI (WT: 1; MADM2: 1,508; MADM3: 1,663); the exact *p*-values are indicated, ns=not significant. Source data are provided as a Source Data file. **(b)** Phalloidin fluorescent staining of actin filaments, together with DAPI, in MADM2, MADM3 and a healthy control (WT) fibroblast cell lines at equivalent culture passages (P11-P13). Data shown is representative of 2 independent experiments. Scale bar: 40µm.

Supplementary figure 10

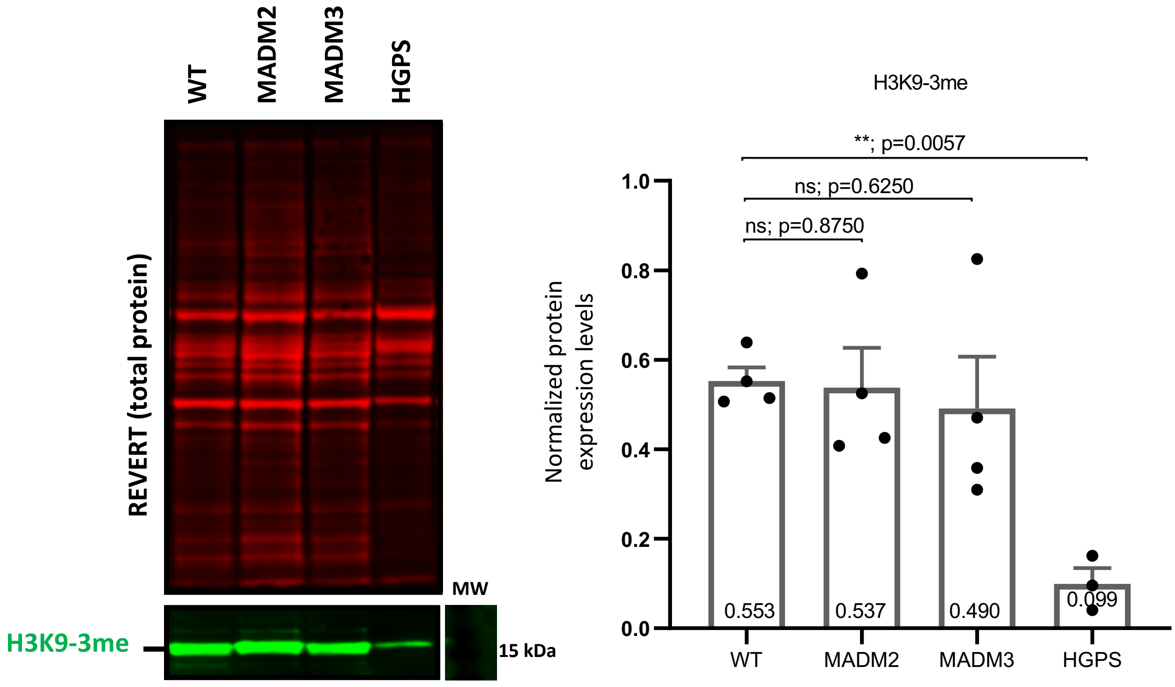

Supplementary Figure 10: quantification of Histone 3-Lysine 9 trimethylation (H3K9-3me) levels. A representative Western blot experiment in fibroblast whole cell lysates from healthy control (WT), MADM2, MADM3 and a HGPS patient, showing H3K9-3me expression. Protein levels were quantified by ImageJ software and their expression levels were normalized to total protein (Revert staining). Results are expressed as mean ± SEM, n=4 independent experiments except for HGPS (n=3); Wilcoxon matched-pairs signed rank test, the mean values are indicated within bars, Lower 95% CI (WT: 0,4569; MADM2: 0,255; MADM3: 0,1205; HGPS: --0,05159), Upper 95% CI (WT: 0,6490; MADM2: 0,8202; MADM3: 0,8613; HGPS: 0,2505); the exact *p*-values are indicated, ns=not significant. **p<0,01. Source data are provided as a Source Data file.

Supplementary Figure 11

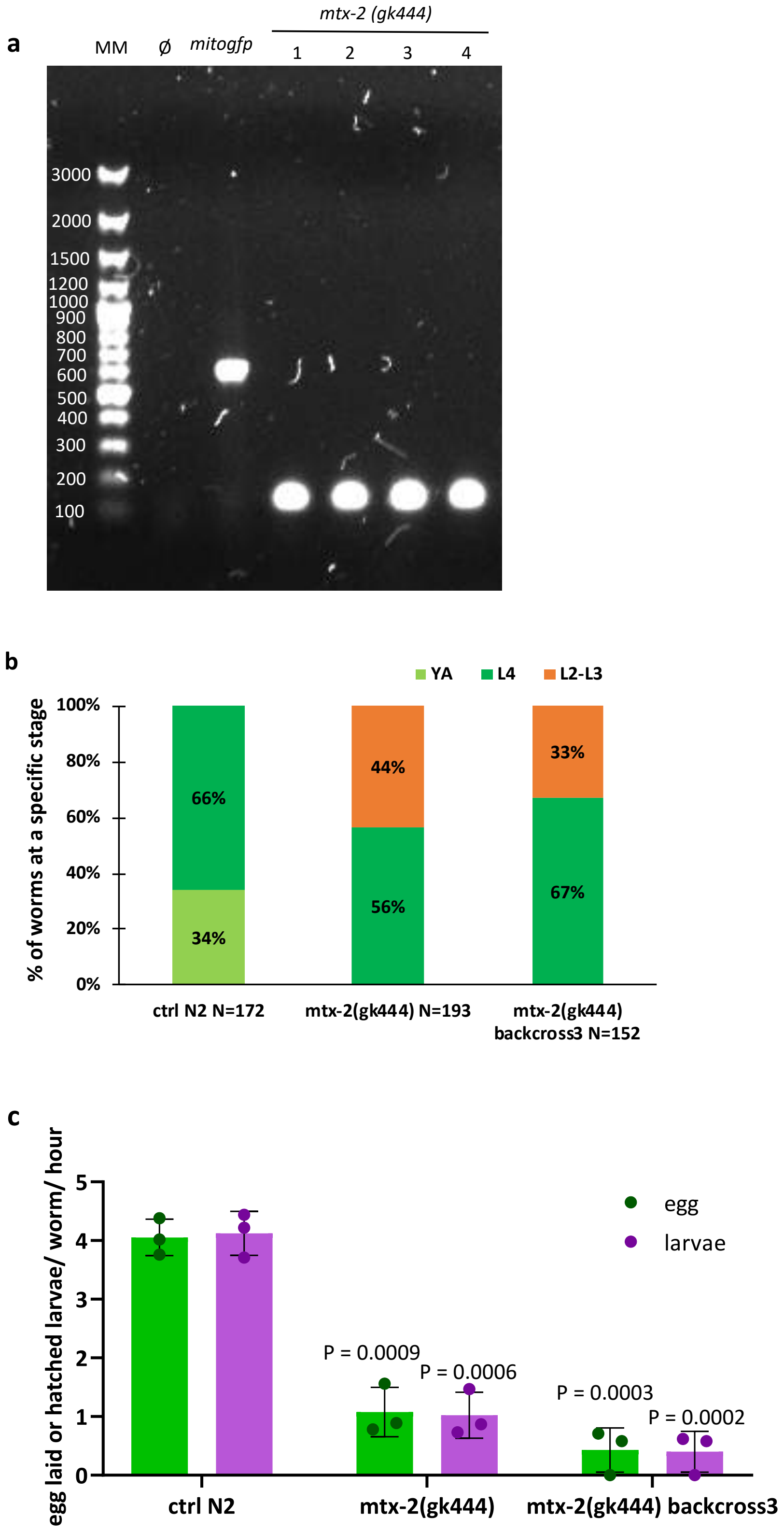

Supplementary Figure 11: characterization of *mtx-2* KO *C. elegans* strain *mtx-2(gk444).* (a) Representative gel image of PCR amplification for genotyping *mtx-2* alleles in *mitogfp* worms and transgenic *ccIs4251; mtx-2(gk444)* clones. Wild type *mtx-2* alleles yield 657 bp (base pairs) PCR products whilst *mtx-2(gk444)* KO alleles yield 132 bp PCR products. MM: GeneRuler^{TM} 100 bp Plus DNA ladder; Ø: negative control; *mitogfp* strain; 1: *mtx-2(gk444)*; 2: *ccIs4251; mtx-2(gk444)* 2.7 clone; 3: *ccIs4251; mtx-2(gk444)* 4.3 clone; 4: *ccIs4251; mtx-2(gk444)* 5.4 clone. The gel image is representative of 5 independent experiments. **(b)** Representative data of developmental assay for N2 (first column), *mtx-2(gk444)* (second column) *and mtx-2(gk444)* backcrossed 3 times on the N2 background (third column). The values within the column represent the percentage of worms at a specific stage from a population (N). L2-L3 and L4: subsequent larval stages, YA = young adult stage. Light green, green and orange bars represent the specific developmental stage of the worms. The data were analyzed from 3 independent experiments. **(c)** Representative data of fertility assay for N2, *mtx-2(gk444)* and *mtx-2(gk444)* backcrossed 3 times on N2 background. Green and purple bars represent the number of laid eggs and the number of hatched larvae, per worm per hour, respectively. Dots depict individual samples. Data are presented as mean ± SD. The *p*-values were obtained by two-tailed unpaired *t* test comparing the mutants' values to the N2 control. The data were analyzed from 3 independent experiments. Source data are provided as a Source Data file.

Supplementary Figure 12

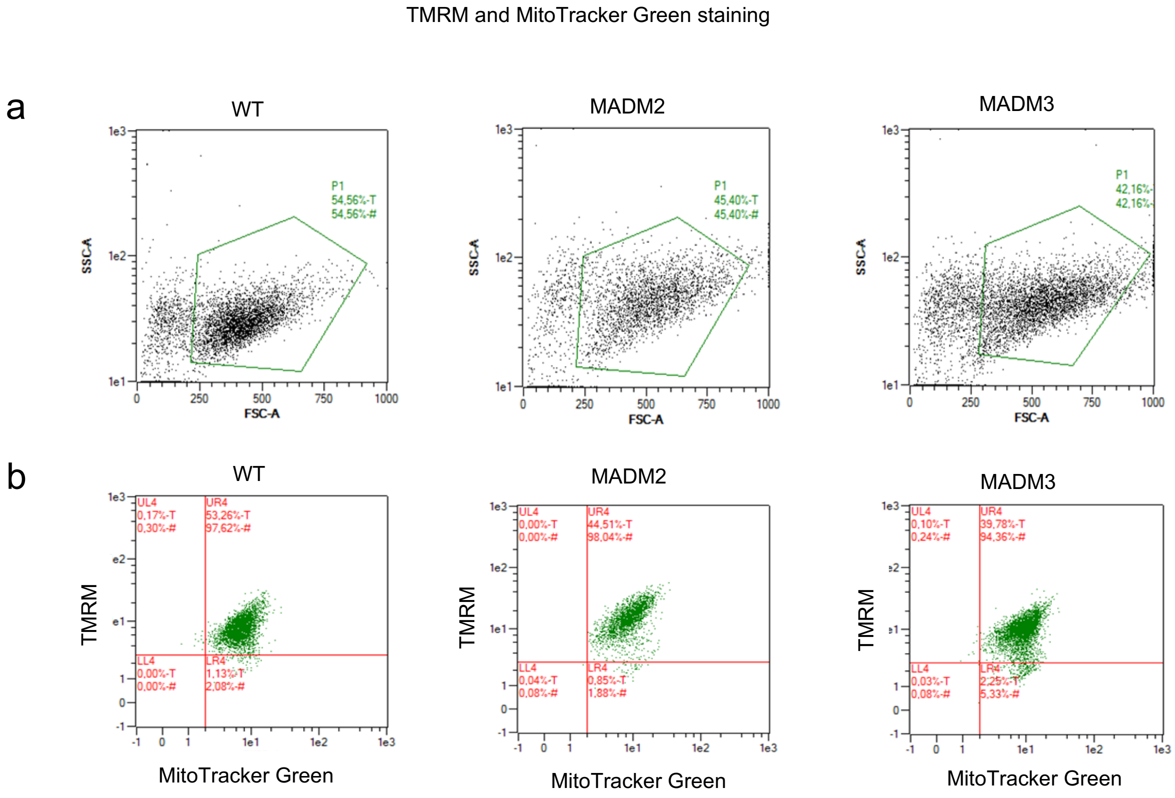

Supplementary Figure 12: FACS sequential gating/sorting strategies relative to Figure 3d.

(a) The gating strategy is presented in the forward and side scatter plots that select the cell population to analyze (P1) and eliminate dead cells from the WT, MADM2 and MADM3 samples. **(b)** The two parameter density plots combine the TMRM (ordinate, dependent of the membrane potential) and Mitotracker Green (abscissa, independent of the membrane potential) relative fluorescence for each cell selected from the P1 cell populations selected in a. Values are the percentage of the P1 populations. The histograms in Figure 3d illustrate the relative values of the TMRM fluorescence.
