# Supplementary material for: Loss of MTX2 causes mandibuloacral dysplasia and links mitochondrial dysfunction to altered nuclear morphology
Source: Nat Commun. 2020 Sep 11;11:4589. doi: 10.1038/s41467-020-18146-9 (PMC7486921; doi:10.1038/s41467-020-18146-9)
Supplement: Supplementary file 3 — Description of Additional Supplementary Files [file 41467_2020_18146_MOESM3_ESM.pdf]

**Description of Additional Supplementary Files**

**File Name**: Supplementary Data 1
**Description:** Additional clinical features of MADaM patients, compared to other progeroid syndromes.
